# The value of combined detection of specific immunoglobulin E, interleukin-6 and regulatory T cells in predicting the risk of postoperative recurrence in patients with eosinophilic chronic rhinosinusitis and nasal polyps

**DOI:** 10.5937/jomb0-48780

**Published:** 2024-06-15

**Authors:** Xudong Gao, Jin Zhang, An Li, Yu Ding, Bo Zhao, Yujuan Wang

**Affiliations:** 1 Shaanxi Provincial People's Hospital, Department of Otolaryngology Head and Neck surgery, Xi'an, China

**Keywords:** specific immunoglobulin E, interleukin-6, regulatory T cells, eosinophilic chronic rhinosinusitis with nasal polyps, postoperative recurrence, risk, forecast, specifični imunoglobulin E, interleukin-6, regulatorne T ćelije, eozinofilni hronični rinosinusitis sa nazalnim polipima, postoperativni recidiv, rizik, prognoza

## Abstract

**Background:**

To investigate the predictive value of specific immunoglobulin E (sIgE), interleukin-6 (IL-6) and regulatory T cells (Treg) on the risk of postoperative recurrence in patients with eosinophilic Chronic rhinosinusitis with nasal polyps (EcRswNP).

**Methods:**

A total of 198 patients with EcRswNP collected to our Hospital from January 2019 to December 2021 were selected as the research subjects. All patients underwent functional endoscopic sinus surgery. The patients were selected to recurrence group (RG, n = 48) and nonrecurrence group (NRG, n = 150) on the basis of the recurrence after 1 year of follow-up. The related factors of postoperative recurrence of EcRswNP were analyzed. The ROC was used to analyze the dangerous of sIgE, IL-6 and Treg in predicting postoperative recurrence of EcRswNP patients.

**Results:**

The proportion of asthma patients, nasal congestion VAS score, and peripheral blood Eos% content in the RG exceeded that in the NRG, and the Organization Neu % and peripheral blood Neu% levels were less than those in the NRGp (P all < 0.05). The serum sIgE and serum IL6 in the RG were higher than those in the NRG, while the level of peripheral blood Treg was lower than that in the NRG (P < 0.05). Logistic regression analysis showed that high levels of serum sIgE, serum IL-6 and low Treg levels were risk factors for postoperative recurrence (P < 0.05). ROC showed that the AUC of peripheral blood sIgE level, IL-6 and Treg levels alone in predicting the dangerous of postoperative recurrence in patients with EcRswNP were 0.786, 0.707 and 0.636, respectively (all P < 0.05); The AUC of combined prediction of peripheral blood sIgE, IL-6 and Treg levels for postoperative recurrence dangerous in patients with EcRswNP was 0.973, indicating that the efficacy of jointed prediction was exceed than that of single prediction (P < 0.05).

**Conclusions:**

The high levels of sIgE, IL6 and low Treg levels in patients with EcRswNP before operation will increase the risk of postoperative recurrence, which is a risk factor affecting postoperative recurrence, and the three indicators have good predictive value for predicting postoperative recurrence in patients with EcRswNP, and the combination of the three indicators has better value in predicting postoperative recurrence.

## Abstract (sr)

**Uvod:**

Cilj je bio da se istraži prediktivna vrednost specifičnog imunoglobulina E (sIgE), interleukina-6 (IL-6) i regulatornih T ćelija (Treg) na rizik od postoperativnog recidiva kod pacijenata sa eozinofilnim hroničnim rinosinusitisom sa nazalnim polipima (EcRsvNP).

**Metode:**

Ukupno 198 pacijenata sa EcRsvNP prikupljenih u našoj bolnici od januara 2019. do decembra 2021. odabrano je kao subjekt istraživanja. Svi pacijenti su podvrgnuti funkcionalnoj endoskopskoj operaciji sinusa. Pacijenti su odabrani u grupu sa recidivom (RG, n = 48) i grupu bez recidiva (NRG, n = 150) na osnovu recidiva nakon 1 godine praćenja. Analizirani su povezani faktori postoperativnog recidiva EcRsvNP. ROC je korišćen za analizu opasnosti od sIgE, IL-6 i Treg u predviđanju postoperativnog recidiva pacijenata sa EcRsvNP.

**Rezultati:**

Udeo pacijenata sa astmom, VAS skor za začepljenost nosa i sadržaj Eos% u perifernoj krvi u RG premašili su onaj u NRG, a nivoi Neu% organizacije i Neu% periferne krvi bili su manji od onih u NRGp (P sve < 0,05). Serumski sIgE i serumski IL-6 u RG su bili viši od onih u NRG, dok je nivo Treg u perifernoj krvi bio niži od onog u NRG (P < 0,05). Logistička regresiona analiza je pokazala da su visoki nivoi serumskog sIgE, serumskog IL-6 i niski nivoi Treg faktori rizika za postoperativni recidiv (P < 0,05). ROC je pokazao da su AUC nivoa sIgE u perifernoj krvi, samo nivoi IL-6 i Treg u predviđanju opasnosti od postoperativnog recidiva kod pacijenata sa EcRsvNP 0,786, 0,707 i 0,636, respektivno (svi P < 0,05); AUC kombinovanog predviđanja nivoa sIgE, IL-6 i Treg u perifernoj krvi za opasan postoperativni recidiv kod pacijenata sa EcRsvNP bio je 0,973, što ukazuje da je efikasnost zajedničkog predviđanja bila veća od efikasnosti pojedinačnog predviđanja (P < 0,05).

**Zaključak:**

Visok nivo sIgE, IL6 i nizak nivo Treg kod pacijenata sa EcRsvNP pre operacije će povećati rizik od postoperativnog recidiva, što je faktor rizika koji utiče na postoperativni recidiv, a tri indikatora imaju dobru prediktivnu vrednost za predviđanje postoperativnog recidiva kod pacijenata. sa EcRsvNP, a kombinacija ova tri indikatora ima bolju vrednost u predviđanju postoperativnog recidiva.

## Introduction

Chronic rhinosinusitis (CRS) is a surgical disease of the ear, nose, throat, head and neck surgery. It is usually mucopurulent nasal discharge, hyposmia or disappearance, head and face oppression or facial pain. The overall prevalence of CRS in China is about 8% which brings great challenges to social economy, medical system and personal quality of life [1]. CRS is usually divided into two types: eosinophilic chronic rhinosinusitis (ECRS) and non-eosinophilic chronic rhinosinusitis (nECRS). Studies have shown that eosinophilic chronic rhinosinusitis with nasal polyps (EcRswNP) is associated with Th2 immune response, and nEcRswNP is associated with Th1/Th17 immune response [2]. Compared with ECRSwNP, nECRSwNP showed different inflammatory manifestations. Nasal polyps are usually benign, originated in the ethmoid sinus, often bilateral onset, located in the bilateral middle meatus, can penetrate into the nasal cavity or sinus cavity. Pathologically, nasal polyps are a manifestation of edema formed by infiltration of a variety of inflammatory cells (neutrophils, plasma cells, etc.). The etiology of CRSwNP is complex and difficult to eradicate, and the impact of conventional drug conservative treatment is not good. Functional endoscopic sinus surgery combined with drug comprehensive treatment is needed. Functional endoscopic sinus surgery has the advantages of clear vision, small trauma, and complete removal of lesions, but the postoperative recurrence rate is high. Glucocorticoid has a high application after endoscopic sinus surgery. After the application of drugs, it can inhibit inflammatory cytokines and further control active neurotransmitter factors, which is conducive to improving the inflammatory response of nasal mucosa and reducing clinical symptoms such as nasal congestion and headache. Although EcRswNP is sensitive to hormone therapy, the duration of efficacy is short, which often leads to repeated delay of the disease [3]. Therefore, it is very important to find serum markers that affect the recurrence of EcRswNP after operation to formulate scientific and reasonable treatment plan and improve the prognosis of patients.

Specific immunoglobulin E (sIgE) plays an important role in the diagnosis of allergic asthma, and has a good diagnostic value for common allergic diseases [4]. Interleukin-6 (IL-6) is involved in the pathological process of many clinical diseases, and is related to the occurrence of bacterial infection, neonatal sepsis and other inflammatory diseases [5]. Regulatory T cells (Tregs) have the function of maintaining immune homeostasis, Studies [6] have found a decrease in the proportion of Treg cells in the tissues of CRSwNP patients. Treg cell injury is considered to be one of the dangerous factors for CRS. Existing studies have shown that [7], the proportion of Treg cells in CRSwNP, especially ECRS patients, is reduced, and there is local immune activation. When the nasal mucosa is stimulated by pathogenic microorganisms to release inflammatory cytokines, the decrease of Treg cells leads to the increase of inflammatory factor synthesis, which is one of the risk factors for CRSwNP. At present, there is no study to explore the clinical value of sIgE, IL-6 combined with Treg cells in predicting the dangerous of postoperative recurrence in patients with EcRswNP. Therefore, this study included 198 patients with EcRswNP, analyzed their clinical data, and explored the predictive value of the combination of sIgE, IL-6 and Treg levels for postoperative recurrence of EcRswNP.

## Materials and methods

### Research object

In this retrospective study, We selected 198 patients with EcRswNP admitted to our Hospital from January 2019 to December 2021 as the research subjects, including 109 males and 89 females, aged 28–67 (47.64 ± 10.08) years. Inclusion criteria : All patients were ≥ 18 years old and met the diagnostic criteria of CRSwNP [8], the main symptoms were nasal congestion, viscous or viscous nasal discharge, and the secondary symptoms were head and face pain, hyposmia or loss of smell; All patients with CT scan or nasal endoscopy showed sinusitis and nasal polyps, peripheral blood eosinophil percentage (Eos %) ≥ 10 % was diagnosed as EcRswNP; All patients underwent functional endoscopic sinus surgery; All patients did not receive antibiotics or hormone therapy within 1 month before surgery. Exclusion criteria: Patients with fungal rhinosinusitis, nasal or paranasal sinus malformation or with malignant tumor of paranasal sinuses; Patients with primary cilia dysfunction or cystic fibrosis; Patients with autoimmune diseases.

### Methods

### General data collection

Collect the patient ‘s gender, age, course of disease, previous history of asthma, allergic rhinitis, smoking and drinking history; Visual Analogue Scales (VAS) were used to evaluate the scores of nasal obstruction, runny nose, olfactory dysfunction and head and facial pain before operation. The scores from no pain symptoms to unbearable pain were recorded as 0–10 points. Eos and neutrophil (Neu) counts in peripheral blood were detected by automatic blood cell analyzer (Hemaray50cRP) before operation, and the percentage was calculated. Histopathological examination of nasal polyps: The specimens of nasal polyps were fixed in 4 % formaldehyde solution. Hematoxylin-eosin staining was used to stain the tissue samples. The number of inflammatory cells, the percentage of Neu and Eos were calculated by 400 times light microscope. Randomly read 10 fields of view, calculate the number of Eos, take the average, and calculate the percentage of Eos.

### Serum sIgE and IL-6 were detected.

Collect 4 mL of peripheral elbow vein blood from all patients upon admission, totaling 2 samples, one of which was centrifuged at 3 500 rpm for 10 min (centrifugal radius 11.5 cm). After separation, take the upper layer of serum and store it at -20°C for examination; another whole blood was stored for inspection. The overall level of serum sIgE was detected by German Allergyscreen, including inhalation allergens including cat hair, dog hair, dander, dust, dust mites, etc. Ingestive allergens include shrimp, crab, milk, mango, pineapple, etc. Serum IL-6 levels were detected by enzyme-linked immunosorbent assay, the reagent kit was from Shanghai Xinyu Biotechnology Co., Ltd.

### Detection of Treg in peripheral blood

BD FACSCanto II flow cytometry was used to detect the level of Treg in peripheral blood. Cell Quest software was used to analyze 10,000 cells, and the results were expressed as percentages. The detection kit was purchased from BD, USA.

### Follow-up evaluation

The nasal cavity was rinsed with 100 mL normal saline in the morning and evening one week after operation, once on each side, and budesonide spray was used for continuous treatment for 6 months. The first follow-up visit was performed 2 weeks after the operation, and the old blood and secretions were removed by nasal endoscopy. The second follow-up visit was performed 1 month after the operation, and the nasal crust and visible vesicles were removed. After that, the patients were followed up for 1 year by outpatient consultation every two months. The follow-up period was up to December 31, 2022, and the postoperative recurrence of all patients was recorded.

### Recurrence evaluation and grouping

The symptoms of the patients were not significantly improved after the operation. One year after the operation, nasal polyps, thick mucus, congestion and edema of the sinus mucosa, and nasal atresia or stenosis were observed by nasal endoscopy [9]. The patients were divided by RG (n = 48) and NRGn (n = 150) on the basis of the recurrence after 1 year of follow-up.

### Statistical analysis

We used Statistic Package for Social Science (SPSS) 26.0 software package (IBM, Armonk, NY, USA). Measurement data as mean ± standard deviation and tested by chi-square (χ^2^); Recorded count data as rate (%) and t test was performed. Logistic regression analysis was used to analyze the related factors of postoperative recurrence of EcRswNP. The ROC was used to analyze the risk of sIgE, IL-6 and Treg in predicting postoperative recurrence of EcRswNP patients. P<0.05 had statistical significance.

## Results

### Comparison of baseline data

The proportion of asthma patients, nasal congestion VAS score and peripheral blood Eos % in the RG were exceed than the NRG, and the Organization Neu % and peripheral blood Neu % were less than the NRG (P < 0.05). See Table 1.

**Table 1:** Comparison of baseline data [(mean±SD)], n (%). Note: EcRswNP: eosinophilic Chronic rhinosinusitis with nasal polyps, VAs: visual analogue scale, Eos %: eosinophil percentage, Neu %: neutrophil percentage

Feature	RG (n=48)	NRG (n=150)	*χ^2^/t value*	*P* value
Gender (male / female)	26/22	83/67	0.020	0.888
Age (years)	47.88±5.63	47.25±6.25	0.620	0.268
Course of disease (years)	3.12±1.41	2.93±1.23	0.882	0.190
Asthma (Yes/No)	28/20	50/100	9.519	0.002
Rhinallergosis (Yes/No)	21/27	46/104	2.780	0.095
Drinking history (Yes/No)	20/28	41/109	3.505	0.061
Smoking history (Yes/No)	19/29	42/108	2.289	0.130
Nasal VAS score	6.94±1.66	6.35±1.71	2.075	0.020
Runny nose VAS score	6.15±1.37	5.88±1.54	1.109	0.134
Olfactory VAS score	5.46±1.54	5.28±1.32	0.780	0.218
Head/face pain VAS score	2.10±0.81	2.05±0.62	0.453	0.326
Organization Eos %	12.16±5.47	11.35±4.26	0.941	0.175
Peripheral blood Eos %	4.47±1.26	3.58±1.04	4.438	<0.001
Organization Neu %	1.32±0.23	1.46±0.57	-2.424	0.008
Peripheral blood Neu %	47.64±6.78	51.49±10.48	-2.965	0.002

### Comparison of sIgE, IL-6 and Treg levels in peripheral blood

The levels of serum sIgE and serum IL-6 in the RG were exceed than the NRG (P < 0.05), The Treg decreased in RG (P < 0.05). See Table 2.

**Table 2:** The levels of sIgE, IL-6 and Treg in peripheral blood of the two groups were (mean ± SD). Note: sIgE: specific immunoglobulin E, IL-6: interleukin-6, Treg: regulatory T cells

Feature	RG (n=48)	NRG (n=150)	*χ2/t value*	*P* value
Serum sIgE (IU/L)	3.26±1.53	1.87±0.35	6.257	<0.001
Serum IL-6 (pg/mL)	18.47±5.32	15.18±2.44	4.131	<0.001
Treg in peripheral blood (%)	4.87±1.16	9.53±2.83	-16.332	<0.001

### Risk factors analysis

The postoperative recurrence of EcRswNP patients was used as the dependent variable (yes = 1, no = 0), and the remaining meaningful indicators as independent variables, among which asthma was a binary variable (assignment: yes = 1, no = 0). Nasal obstruction VAS score, peripheral blood Eos %, peripheral blood Neu %, serum sIgE, serum IL-6 and peripheral blood Treg were continuous variables, and the assignment was included in the actual value. Logistic regression analysis showed that high serum sIgE, IL-6 and low Treg levels were hazard factors for postoperative recurrence (P < 0.05). See Table 3.

**Table 3:** Analysis of risk factors affecting postoperative recurrence in patients with EcRswNP

Variable	β	SE	Wald χ2	P	OR (95%CI)
Nasal VAS score	0.306	0.221	1.914	0.166	1.358 (0.880~2.094)
Peripheral blood Eos %	1.109	0.435	6.494	0.011	3.031 (1.292~7.111)
Peripheral blood Neu %	-0.062	0.041	2.278	0.131	0.940 (0.867~1.019)
Serum sIgE	1.555	0.434	12.816	<0.001	4.733 (2.021~11.087)
Serum IL-6	0.388	0.123	9.986	0.002	1.474 (1.159~1.875)
Treg in peripheral	-1.130	0.266	17.997	<0.001	0.323 (0.192~0.544)
Constant	-7.001	4.217	2.756	0.097	–

### The ROC curve predicted separately

**Figure 1:**
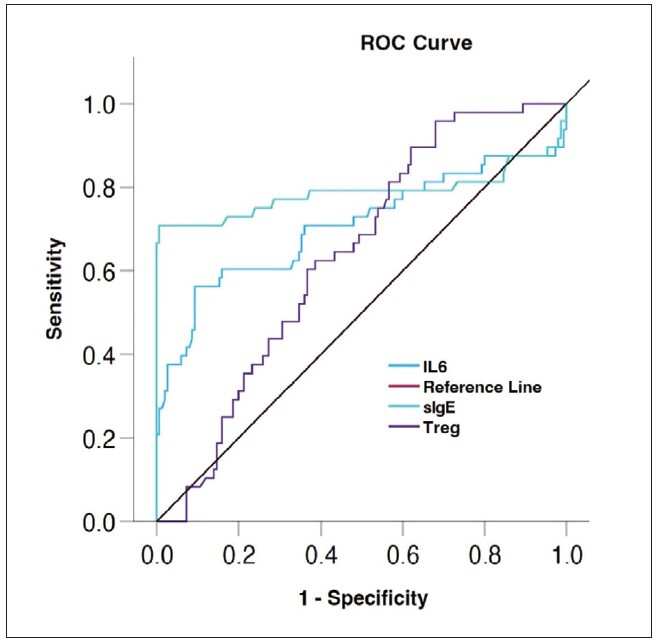
Hologram technology can specifically observe renal blood vessels, renal pelvis and renal lamps.

ROC showed (Figure 1), the AUC of peripheral blood sIgE level, IL-6 and Treg level alone in predicting the hazard of postoperative recurrence in patients with EcRswNP were 0.786,0.707 and 0.636, respectively (P all < 0.05). See Table 4.

**Table 4:** ROC prediction efficiency

Index	AUC	Specificity	Sensitivity	95%CI	Youden index
Serum sIgE	0.786	0.887	0.708	0.681~0.892	0.595
Serum IL-6	0.707	0.907	0.563	0.605~0.810	0.470
Treg in peripheral	0.636	0.380	0.891	0.556~0.716	0.271
Joint prediction	0.973	0.973	0.896	0.945~1.000	0.869

### ROC curve of Joint prediction

**Figure 2:**
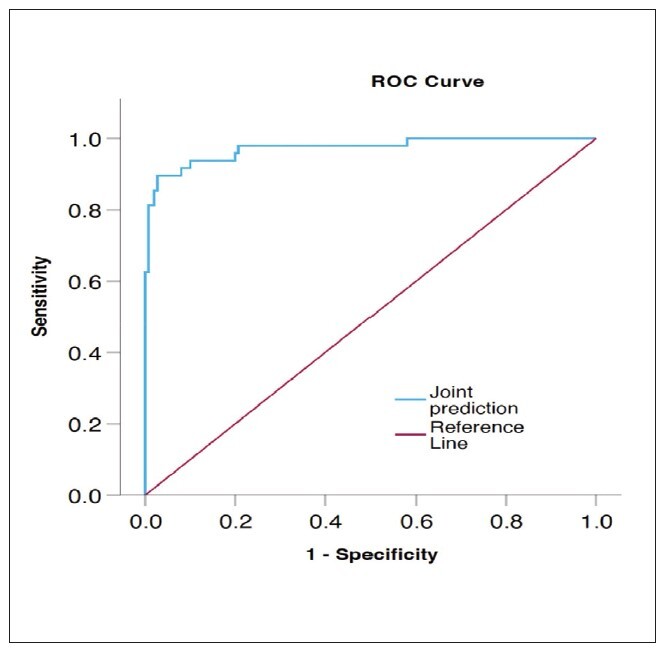
Preoperative evaluation of stones, renal pelvis, and renal calyx and selection of puncture target lamps.

The AUC of peripheral blood sIgE, IL-6 and Treg levels combined to predict the dangerous of postoperative recurrence in patients with EcRswNP was 0.973 (Figure 2). And the performance of joint prediction is very good (P < 0.05).

## Discussions

EcRswNP is a heterogeneous and inflammatory disease, which is difficult to treat and has the characteristics of repeated attacks, causing serious interference to patients‘ daily life and work [10]. In clinical practice, treatment for EcRswNP primarily involves surgery combined with comprehensive drug therapy. Incomplete surgical treatment may lead to nasal polyp recurrence. Some patients still experience recurrence despite radical treatment. Therefore, it has been deeply concerned in clinical practice. The etiology of EcRswNP is complex, and it is mostly considered to be the result of a variety of factors, among which inflammatory factors play an important role. Inflammatory factors can promote the inflammatory response, change the local microenvironment of the nasal cavity, promote the formation of nasal polyps, and increase the risk of postoperative recurrence. According to research reports, the recurrence of CRSwNP is closely related to factors such as peripheral blood Eos, sIgE, pro-inflammatory factors IL-6 and Treg [11] [12]. This article explores the effects of sIgE, pro-inflammatory factors IL-6 and Treg on postoperative recurrence in patients with EcRswNP, and predicts the risk of postoperative recurrence.

Eosinophils are secondary effector cells involved in the pathogenesis of allergic reactions and multifunctional immune inflammatory cells. This feature is both beneficial and harmful to the development of CRSwNP. In the development of CRSwNP, eosinophils are activated when they are recruited to nasal polyps. The activated eosinophils secrete a large number of factors, which will cause type 2 inflammatory response, resulting in increased local eosinophils in the tissue and aggravating the inflammatory response. These factors contribute to a type 2 inflammatory response, resulting in an increase in local eosinophils within the tissue and exacerbating the inflammatory response. In this study, automatic blood cell analyzer (Hemaray50cRP) was used to detect peripheral blood eosinophils (Eos) and neutrophils (Neu) counts. Eos and Neu counts were read at high magnification and their percentages were calculated with relatively high accuracy. Current clinical studies use this method to calculate Eos % levels. Studies [13] have found that eosinophils are also associated with the currence of nasal polyps. Eso % elevation in peripheral blood was also associated with nasal polyps, and patients with a percentage of peripheral blood eosinophils greater than 3.8% are more than twice as likely to have recurrence of nasal polyps as patients with a percentage of peripheral blood eosinophils less than 3.8 %. Studies [14] have found that compared with a single increase in peripheral blood or tissue eosinophils, a simultaneous increase in both increases the probability of nasal polyps. In addition, studies [15] have found that the proportion of eosinophils in nasal polyps more than 27% or the absolute count of eosinophils more than 55 per high magnification is the strongest predictor of postoperative recurrence of nasal polyps in the Chinese population.

In this study, we conducted a 1-year follow-up observation. The postoperative recurrence rate of EcRswNP patients was 24.24% (48/198). The proportion of asthma patients, nasal congestion VAS score, and peripheral blood Eos % in the RG were exceed than the NRG. It shows that many factors may cause disease recurrence in patients with EcRswNP after surgical treatment. In order to further analyze the main factors affecting postoperative recurrence in patients with EcRswNP. In this study, the logistic regression analysis model to show that high levels of sIgE, IL-6 and low Treg levels were dangerous factors for postoperative recurrence in patients with EcRswNP. The possible reason is that the allergen enters the body for the first time, which will be quickly recognized by the immune system and initiate the immune mechanism. Plasma cells secrete sIgE into the blood, sIgE binds to immune cell receptors to complete the sensitization reaction, and activates a series of immune processes. The release of inflammatory factors promotes the formation of nasal polyps, and increases the risk of postoperative recurrence [16]. Studies have found that [17], the level of fungal sIgE in patients with EcRswNP showed an increasing trend, suggesting that fungal sIgE was significantly related to the pathological characteristics of patients with EcRswNP. Serum IL-6 is a member of the proinflammatory cytokine family, Elevated serum IL-6 level indicates inflammatory reaction. Studies have shown that [18], serum IL-6 is high in peripheral blood of patients with CRS. Elevated IL-6 inhibits Rasmitogen protein activating enzyme, thereby inducing local inflammatory response and increasing the risk of EcRswNP. According to reports [19], Treg can inhibit the function of eosinophils. Treg is mediated by interleukin-10 (IL-10) to inhibit the mechanism of eosinophil activation, and may also control allergic reactions by inducing neutrophil apoptosis or promoting these cells. By mediating immune regulation and immune tolerance, Treg cells can maintain a stable internal environment and inhibit inflammatory responses by mediating immune regulation and tolerance. Under normal circumstances, effector T cells and Treg cells balance each other and maintain immune tolerance together. However, when the level of Treg cells decreases, it may weaken the inhibitory effect on effector T cells, increase the release of interleukin 4 and other inflammatory mediators, and aggravate pro-inflammatory response, thus inducing the dangerous of postoperative recurrence in patients with EcRswNP [20].

The ROC curve can be used to predict the risk of disease. The AUC is between 0.1 and 1. The higher the AUC value, the better the prediction model can distinguish between high and low risk groups. In this study, ROC showed that the AUC of peripheral blood sIgE level, IL-6 and Treg level alone in predicting the dangerous of postoperative recurrence in patients with EcRswNP was 0.786, 0.707 and 0.636, respectively, indicating that sIgE level, IL-6 and Treg level alone had good predictive efficacy. Further analysis showed that the AUC of peripheral blood sIgE, IL-6 and Treg levels in predicting the dangerous of postoperative recurrence in patients with EcRswNP was 0.973. This result shows that the efficiency of joint prediction is exceed than that of individual prediction, and joint prediction has better efficiency and exceed predictive value. Limitations of this study: Due to the limitation of time and sample size, the selected subjects are all from our hospital; in the later stage, the sample size can be expanded clinically, or a multi-center study can be conducted to prolong the follow-up time, so as to further explore the clinical value of sIgE, IL6 and Treg for postoperative recurrence in patients with EcRswNP.

## Conclusion

In summary, preoperative high levels of sIgE, IL6 and low Treg levels in patients with EcRswNP will increase the risk of postoperative recurrence, which is a risk factor affecting postoperative recurrence, and the three indicators have a good predictive value for predicting postoperative recurrence in patients with EcRswNP, and the combination of the three indicators has a better predictive value for postoperative recurrence.

## Dodatak

### Conflict of interest statement

All the authors declare that they have no conflict of interest in this work.
